# How immediate and significant is the outcome of training on diversified diets, hygiene and food safety? An effort to mitigate child undernutrition in rural Malawi

**DOI:** 10.1017/S1368980017003652

**Published:** 2018-01-17

**Authors:** Anitha Seetha, Takuji W Tsusaka, Timalizge W Munthali, Maggie Musukwa, Agnes Mwangwela, Zione Kalumikiza, Tinna Manani, Lizzie Kachulu, Nelson Kumwenda, Mike Musoke, Patrick Okori

**Affiliations:** 1 International Crops Research Institute for the Semi-Arid Tropics (ICRISAT), PO Box 1096, Lilongwe, Malawi; 2 Lilongwe University of Agriculture and Natural Resources (LUANAR), Lilongwe, Malawi

**Keywords:** Undernutrition, Complementary feeding, Malnutrition, Randomised controlled trial

## Abstract

**Objective:**

The present study examined the impacts of training on nutrition, hygiene and food safety designed by the Nutrition Working Group, Child Survival Collaborations and Resources Group (CORE).

**Design:**

Adapted from the 21d Positive Deviance/Hearth model, mothers were trained on the subjects of appropriate complementary feeding, water, sanitation and hygiene (WASH) practices, and aflatoxin contamination in food. To assess the impacts on child undernutrition, a randomised controlled trial was implemented on a sample of 179 mothers and their children (<2 years old) in two districts of Malawi, namely Mzimba and Balaka.

**Settings:**

A 21d intensive learning-by-doing process using the positive deviance approach.

**Subjects:**

Malawian children and mothers.

**Results:**

Difference-in-difference panel regression analysis revealed that the impacts of the comprehensive training were positive and statistically significant on the *Z*-scores for wasting and underweight, where the effects increased constantly over time within the 21d time frame. As for stunting, the coefficients were not statistically significant during the 21d programme, although the level of significance started increasing in 2 weeks, indicating that stunting should also be alleviated in a slightly longer time horizon.

**Conclusions:**

The study clearly suggests that comprehensive training immediately guides mothers into improved dietary and hygiene practices, and that improved practices take immediate and progressive effects in ameliorating children’s undernutrition.

Growth impairment experienced by infants and young children due to deficiency and imbalance in nutritional intake has been a major problem in low-income countries. In particular, sub-Saharan Africa registers high incidences of child stunting and underweight^(^ ^1^ ^)^. Malawi is one of the least developed nations, where the recent rates of stunting, underweight and wasting are 42, 17 and 4 %, respectively^(^ ^2^ ^)^.

Wasting, stunting and underweight are the three popular indicators of undernutrition. Wasting can be caused by an extremely low energy intake (e.g. due to famine), nutrient losses due to infection, or a combination of both. Caregivers and health providers can sometimes contribute to wasting if the child is placed on an improper diet^(^ ^3^ ^,^ ^4^ ^)^. Stunting is multifactorial impairment in linear growth that results from undernutrition, recurrent infections with water-borne diseases, substandard health care and environmental enteropathy due to improper sanitation^(^ ^5^ ^,^ ^6^ ^)^. Underweight is a composite measure of wasting and stunting, and is regarded as the overall extent of undernutrition among children^(^ ^7^ ^)^.

The most direct cause for child undernutrition is deficiency in macro- and micronutrients. Diets dominated by staples tend to lead to deficiency in protein, fat and micronutrients, particularly the amino acid lysine^(^ ^8^ ^)^. In fact, a survey by the Malawi Vulnerability Assessment Committee revealed that the starchy cereals, primarily maize, account for more than 70 % of daily energy intake in Malawi^(^ ^9^ ^)^. By contrast, intake of legumes provides access to all nine essential amino acids, as well as the B-vitamin group, ascorbic acid and carotene^(^ ^10^ ^)^. Pigeon pea, for instance, has a protein content of 21–26 %^(^ ^11^ ^)^. Small grain cereals like finger millet, for example, contains 340 mg Ca/100 g, three times the concentration in milk^(^ ^12^ ^)^, is gluten-free and easily digestible by the human body. Legumes and small grain cereals together would complement maize-based diets and help to achieve improved health and growth outcomes. Notwithstanding the availability of crops in Malawi, utilisation of such complementary food ingredients, especially finger millet and pigeon pea, remains low on average due to food consumption traditions and limited awareness of the nutrient content and its health benefits. Diets among smallholders in rural Malawi have traditionally been dominated by a single staple crop, which is maize in most parts of the country and sorghum to a lesser extent^(^ ^13^ ^)^. Women cook hard porridge called *nsima* from maize and the family consumes it at home^(^ ^14^ ^)^. Rural households tend to produce such crops as finger millet and pigeon pea for other purposes such as brewing and trading for economic benefits. Higher consumption of such crops would contribute to enhancing dietary diversification and better nutritional outcomes.

Another important factor affecting child growth is food safety. Aflatoxin (*Aspergillus flavus*) is a harmful mycotoxin that contaminates a variety of food crops^(^ ^15^ ^)^. It alters the cellular and biochemical functions of the intestine, affects intestinal architecture, inhibits intestinal regeneration, triggers Zn deficiency^(^ ^1^ ^,^ ^5^ ^)^ and hinders vitamin A absorption^(^ ^16^ ^)^. Chronic aflatoxin exposure impairs child growth^(^ ^17^ ^)^ by restraining dietary nutrient uptake^(^ ^18^ ^)^ and suppressing immunity, which leads to susceptibility to endemic diseases^(^ ^19^ ^)^. Forty-five per cent of maize samples from Malawi were contaminated with more than 4 ppb of aflatoxin, the proposed upper limit for human consumption^(^ ^20^ ^)^. Likewise, 46 and 23 % of groundnut samples collected in Malawi in 2008 and 2009, respectively, were contaminated^(^ ^21^ ^)^. Improper pre- and post-harvest crop management is the primary factor causing high levels of aflatoxin contamination in crops^(^ ^22^ ^,^ ^23^ ^)^. Aflatoxin levels in man decrease with the degree of dietary diversity and dependence on single staple foods exposes consumers to aflatoxin hazard^(^ ^24^ ^)^. Moreover, no cases of severe aflatoxin contamination in finger millet and pigeon pea crops have been reported thus far.

In addition to nutrition diversification and aflatoxin control, water, sanitation and hygiene (WASH) practices also play a fundamental role in improving health outcomes. The WHO estimates that 50 % of undernutrition cases are associated with repeated diarrhoea or intestinal worm infections caused by unsafe water, poor sanitation and insufficient hygiene^(^ ^25^ ^)^.

Our intervention programme adopted the Positive Deviance (PD)/Hearth model, which is a method of conducting training of mothers through group activities in contrast to individual counselling. The method was developed and employed in the 1980s^(^ ^26^ ^)^ and was replicated in later years. In this approach, ‘positive deviant’ (PD) mothers living in resource-poor conditions who are raising healthy and well-nourished children are selected to lead sessions attended by small groups of undernourished children and their mothers living in the same resource-poor conditions^(^ ^27^ ^)^. The aim is to find out the PD mothers’ practices that allow their children to be well nourished; for example, the way complementary food is used. Community health practitioners can then incorporate these PD behaviours into their nutrition programmes, knowing that the behaviours are both affordable and culturally acceptable since they are already being practised by members of the community^(^ ^28^ ^)^. In different programmes around the world, the PD/Hearth method has worked to improve health habits, enhance nutrition knowledge and consequently rehabilitate undernourished children^(^ ^29^ ^,^ ^30^ ^)^. In Malawi, a few non-governmental organisations such as GOAL and I-LIFE have successfully adopted the PD/Hearth approach in their nutrition programmes to date, although quantitative impact assessment has not been conducted^(^ ^31^ ^,^ ^32^ ^)^.

Most of the previous nutrition intervention programmes incorporating the PD/Hearth model entailed qualitative assessment or, if quantitative, descriptive statistics such as mean/percentage and comparison tests (e.g. *t* test, *χ* ^2^ test). Those analyses are useful enough to derive strong qualitative implications. However, to quantify the programme effects on undernutrition indicators with minimised estimation bias, it is imperative to employ a more sophisticated method of quantification of impacts. For this purpose, the present study utilised a combination of the randomised controlled trial and panel regression analyses. Another unique aspect of our assessment is that the undernutrition indicators were measured four times during the 21d intervention programme, including the baseline and endline, which enabled us to monitor how the effects on different indicators evolved during the programme period. As such, the present article focuses on the immediate effects of the intervention rather than the long-term consequences, which will be studied in a separate article.

While it is widely believed that tangible progress in improving nutritional outcomes and child health can be obtained through better nutrition knowledge^(^ ^33^ ^)^, improved feeding practices^(^ ^34^ ^)^, hygiene^(^ ^35^ ^)^ and food safety management^(^ ^36^ ^,^ ^37^ ^)^, formulation of an integrated approach to transforming conventional practices into improved ones would be accelerated with accumulated scientific and empirical evidence from the ground.

The objective of the present study was to evaluate the effectiveness of a training programme that combined four elements from different initiatives around the world: (i) dietary diversification using locally produced affordable food; (ii) WASH practices; (iii) food safety management, specifically aflatoxin control; and (iv) the PD/Hearth model as a method of information sharing. Furthermore, the effectiveness was quantified by methods beyond descriptive statistics.

## Methodology

### Household survey

The survey was undertaken during the 2014/15 post-harvest storage period. There is one rainy season in Malawi that usually ends in April. Although food availability is better during the post-harvest than during the pre-harvest season, aflatoxin contamination is more salient in the former due to smallholders’ unfavourable storage environments. Water sanitation is also worse during the dry season^(^ ^38^ ^)^.

Purposive sampling was used to select districts based on the extent of: (i) additional crop (especially legume crops) production other than maize; (ii) aflatoxin contamination in groundnut and maize^(^ ^21^ ^)^; and (iii) the stunting rate. Two hundred and forty-one mothers/caregivers who had children aged 6–23 months who had started consuming complementary food, had no congenital disorder and were capable of swallowing complementary food were randomly recruited from rural communities in Mchinji (*n* 41), Balaka (*n* 100) and Mzimba (*n* 100) districts.

A semi-structured questionnaire was converted into the ODK (Open Data Kit) form to electronically capture the household-level data on demographic and socio-economic characteristics, knowledge on complementary feeding, WASH, aflatoxin awareness, ingredients for complementary foods, agricultural practices, and infant and young child feeding practices, to establish the current knowledge, attitudes and practices towards hygiene, aflatoxin and nutrition. Seven types of dietary indices were also collected in accordance with the Household Dietary Diversification Score (HDDS)^(^ ^39^ ^,^ ^40^ ^)^. Grain samples of maize, groundnut and sorghum were collected for aflatoxin assay, while urine samples were collected from the children to assess levels of the aflatoxin M1 (AFM1) biomarker.

### Randomised controlled trial

The training and follow-up measurements were conducted in Mzimba and Balaka districts. Among the 200 households in these districts at baseline, 179 households that explicitly showed interest participated in the randomised controlled trial whereby ninety-one households (forty-six from Mzimba and forty-five from Balaka) were randomly assigned to the intervention group and the remaining eighty-eight to the control group. In the study, there was one intervention group that received the intervention programme and one control group that did not. On the day before the start of the trial (i.e. day 0), anthropometric measurements were collected from the children and their 24 h dietary recall was also recorded. During the 21d trial, information on disease incidences and food acceptability was collected every day, while follow-up anthropometric measurements were registered on day 7, day 14 and day 21. The contents and schedule of the intervention programme can be found in the ‘Training programme’ subsection below.

### Indicators of child undernutrition

The *Z*-scores for wasting, stunting and underweight^(^ ^41^ ^)^ were adopted as main indicators of child undernutrition. Anthropometric measurements were conducted on all children participating in the study. Their height, weight and age were used to calculate their weight-for-height *Z*-score (wasting), height-for-age *Z*-score (stunting) and weight-for-age *Z*-score (underweight), which were calculated using WHO Anthro software version 3.2.2. Children were categorised as severely undernourished, moderately undernourished and overnourished if the corresponding *Z*-score was below −3·0, between −3·0 and −2·0, and above +2·0, respectively. *Z*-scores between −2·0 and +2·0 indicate normal nutritional status. Additionally, mid-upper arm circumference (MUAC), which indicates the muscle mass of the upper arm, was also measured. According to the WHO standard, wasting and MUAC are considered as criteria for severe acute undernutrition, while stunting indicates chronic undernutrition^(^ ^42^ ^)^.

The children’s weight was measured using calibrated Salter scales. To measure their height, recumbent length measurements were adopted using a stadiometer. MUAC was measured to the nearest 0·1 cm using a MUAC tape wrapped around the mid-upper arm.

### Crop sample collection and aflatoxin B1 assay

One hundred grams of maize grains that would have been used for complementary food preparation were collected from each of the sampled households for aflatoxin detection. During sample collection, a representative sample was obtained by mixing ten sub-samples, each weighing 10 g and collected from different parts of each storage bag, to constitute a sample of 100 g that was used to assay for aflatoxin contamination. The 100 g samples were weighed and powdered finely, and then two replicate samples of about 20 g each were mixed with 100 ml of 70 % methanol (v/v) containing 0·5 % potassium chloride (w/v) and were blended further. The mixture was then transferred to a 250 ml conical flask, shaken at 300 rpm for 30 min (Gallenkamp Orbital Shaker, CAT # SCM 300 0101, UK) and filtered through Whatman No. 41 filter paper (GE Healthcare, UK). The filtrate was then analysed using indirect competitive ELISA using a ninety-six-well ELISA plate (F96 MAXISORP; Thermo Fisher Scientific, Denmark) as indicated earlier^(^ ^21^ ^)^. In brief, the samples were tested using polyclonal antibody produced against aflatoxin B1–bovine serum albumin conjugate. Alkaline phosphatase-conjugated anti-rabbit antibodies (Sigma-Aldrich, USA) were used as a secondary antibody and *p*-nitrophenyl phosphate (Sigma-Aldrich) was used as a substrate. The colorimetric reaction was measured in an ELISA plate reader (Multiscan Reader; Thermo Fisher Scientific, China) at 405 nm.

### Urine sample collection and aflatoxin biomarker assay

Each mother in the study was provided with urine sample collection vials and was advised to collect early-morning urine samples of her child. These vials were collected from the mothers immediately and transported to the ICRISAT laboratory in an ice box. The urine samples were stored at −20°C until use. The analysis was performed using a commercial ELISA kit (Sigma-Aldrich) according to the manufacturer’s instruction. The urine samples collected from children were given lab numbers and filtered, and the clear solution was taken for the analysis. The AFM1 standards provided in the kit (0·0, 0·15, 0·40, 0·8, 1·5 and 4 ng/ml) and the urine samples collected from the children were aliquoted 1:20 (50 µl+950 µl distilled water) into 2 ml microfuge tubes. Then 100 µl of each sample/standard was added to mix wells (provided in kit) with 200 µl of assay buffer, using the multichannel micropipette three to five times. Then 100 µl was taken from each well and added to AFM1 antibody-coated wells of the ELISA plate provided in the kit. After an hour of incubation at 37°C, the plate was washed three times with phosphate-buffered saline–Tween. One hundred microlitres of horseradish peroxidase conjugate was added to each well, incubated for 15 min and washed with phosphate-buffered saline–Tween. Finally, 100 µl of the tetramethylbenzidine substrate was added to each well and stored in a dark place for 15 min. The reaction was stopped by adding stop solution, the yellow colour intensity was read using the plate reader at 450 nm, and then the urine AFM1 concentration was calculated by plotting against the standards.

### Training programme

The PD/Hearth model was adopted for delivering education to mothers on dietary diversification, WASH practices and aflatoxin control in the rural setting. The key steps for this approach were followed from the PD/Hearth programme manual produced by the Nutrition Working Group, Child Survival Collaborations and Resources Group (CORE)^(^ ^43^ ^)^ and were modified according to the context of the locality. During the 21d training period, all mothers and their children from the treatment group gathered at a central place. The nutrition extension staff from the districts participated as facilitators to share with the mothers the importance of different micro- and macronutrient contents in complementary feeding, good hygienic practices, how to choose quality grains to reduce aflatoxin exposure, and how to formulate and prepare porridge from diversified food groups using locally available ingredients. The training was participatory and hands-on, whereby all the mothers were grouped into small groups consisting of six to eight mothers and on each day the porridge was prepared in each group. Each group of mothers took charge of the preparation of complementary food while ensuring that all the appropriate practices were followed by their fellow participants properly. Sessions were conducted for 3 h each day and at the end of each day the mothers were also provided with the complementary flour mix to prepare food at home. The mothers’ practices at home were documented in detail. Leftover food was recorded, if any. Observations were also made to check how the water was treated and how they stored the food and beverages.

More specifically, the following activities were undertaken in the treatment group.

1. Hearth sessions were conducted over 21d by teaching complementary food preparation and feeding, hygiene and food safety modules. During the sessions, small groups of mothers with undernourished children met with PD mothers (so-called ‘volunteer facilitators’) in one of their homes, where they jointly prepared complementary food for their children and fed them.
2. Preparation of complementary porridge consisting of pigeon pea, finger millet, groundnut, maize, carrot and amaranthus leaves by incorporating good feeding practices in the community. The proportions were calculated to provide the children with the required amount of protein, fat and carbohydrate, and the essential micronutrients including vitamin A, Zn, Fe and Ca at the right frequency.
3. The mothers received hands-on experience in cooking recipes formulated for the children throughout the training period. The recommended recipes are presented in Table 1. Sensory evaluation was conducted to choose one recipe over the other.
4. For the preparation of complementary flour, volunteer mothers were trained to ensure that they used good-quality grains by following appropriate steps, including grading the grains based on the quality. The mothers were also educated on the ill effects of aflatoxin and how best to avoid contamination. They were taught post-harvest crop handling methods: segregating damaged and rotten grains, identifying good-quality grains, and grading and storing them appropriately.
5. The volunteer mothers were trained to monitor other mothers for good hygiene practices during feeding: use of boiled water for drinking and cooking, washing hands before preparation/feeding and after using the toilet, washing utensils after preparation and feeding, washing vessels before cooking, and maintaining cleanliness of the surroundings. Thereafter, the volunteer PD mother recorded hygiene practices that were followed by other mothers each day.

**Table 1:** Complementary food recipes recommended for the intervention programme to mitigate child undernutrition in rural Malawi

Recipe 1			Recipe 2		
Crop	Food group	Quantity	Crop	Food group	Quantity
Maize	Cereal	60 g	Maize	Cereal	25 g
Groundnut	Legume	30 g	Finger millet	Cereal	25 g
Carrot	Vitamin A-rich vegetable	½ cup	Groundnut	Legume	25 g
Amaranth	Green leafy vegetable	1 cup	Pigeon pea	Legume	15 g
			Amaranth	Green leafy vegetable	1 cup
			Carrot	Vitamin A-rich vegetable	½ cup

### Analytical methods

To quantify the effects of the training, regression analysis was employed, which allowed us to control for covariates and to obtain standard errors for the treatment effect. Furthermore, our sampling design enabled us to adopt the difference-in-difference model, which is a capable tool to estimate treatment effects by systematically comparing the pre- and post-treatment differences in the outcome between a treatment group and a control group. The difference-in-difference estimation can be utilised only in the presence of multiple-period data sets^(^ ^44^ ^,^ ^45^ ^)^. Moreover, in our case, unobservable time-invariant household characteristics (i.e. fixed effects) can be controlled for since household-level panel data were available, which is a remarkable advantage in minimising estimation bias^(^ ^46^ ^)^. In our study, there are four time points: i.e. *t*=day 0, day 7, day 14 and day 21. Therefore, there are three difference-in-difference dummy variables corresponding to day 7, day 14 and day 21, with day 0 being the base.

The most important assumption with the difference-in-difference estimation is the parallel trends assumption. Provided this assumption holds and we can credibly rule out any other heterogeneous changes over time that may confound the treatment, then the estimators are highly reliable. This assumption holds better when the measurement interval is short, which is the case with our study. All the quantitative analyses, both regression and descriptive statistics, were handled with the statistical software package STATA version 14.

## Results

### Current practices

#### Socio-economic profile and farming practices

The sampled children had an equal gender balance, with the mean age being 8·5 months. Accordingly, the mean age of the sampled mothers was 26 years. Sixty-nine per cent of the households earned their living from farming as the main occupation. The mean asset holding was about $US 20. All sampled farmers were smallholder farmers producing maize for household consumption purposes. The second and third most common crops were groundnut and soyabean, grown for both consumption and sale. The mean landholding size was 0·77 ha (1·9 acres). Most of the farmers did not grade their grain during post-harvest crop handling and storage. Only 23 % of maize producers and 12 % of groundnut producers graded their respective grains. Among those who graded, there were several uses of grade-outs in different forms (Table 2).

**Table 2:** Baseline agricultural practices of the studied households in Mchiniji, Mzimba and Balaka districts, rural Malawi, 2014–2015

Variable/category	No. of households/ total	Frequency (%)
No. of farmers who participated	166/240	69
Grading		
Groundnuts graded	73/166	44
Maize graded	38/166	23
Grading criteria		
Based on rottenness	9/73	12
Based on size and shape	64/73	88
Fate of grade-outs		
Consume in roasted form (mainly groundnuts)	30	42
Consume in the form of peanut butter	24	34
Consume by making into powder (maize to prepare *nsima* which is a porridge)	24	64
Discard (groundnut)	9	13
Discard (maize)	5	13
Feed to livestock		
Groundnut	8	11
Maize	9	23

#### Food consumption practices

Most (96 %) of the sampled children were still fed with breast milk. Eighty-eight per cent of the sampled children had started consuming complementary food with maize porridge. One-third of the children had started consuming complementary food before 6 months of age. Less than a quarter of the mothers had knowledge of the importance of nutrients in complementary food. The proportion of children meeting the minimum dietary diversity was 7 %, with a mean dietary diversity score of 2·0 (sd 1·0), and the proportion of children meeting the minimum meal frequency was 50 %. The dietary diversity score suggested that most children were fed with staples (maize) and vegetables (green leafy) within the last 24 h before the day of the baseline assessment, while other food groups were inadequately consumed, resulting in the composite scale mean score of 2·0 (sd 1·0) out of the full score of 7·0 (Table 3).

**Table 3:** Baseline indicators of dietary habits of infants and young children in the sampled households in Mchiniji, Mzimba and Balaka districts, rural Malawi, 2014–2015

Indicator	Value	Age (months)
Exclusively breast-fed (%)	97	<6 months
Dietary diversity score (max. 7·0)		6–23 months
Mean	2·0	
sd	1·0	
Met minimum dietary diversity (%)	7	6–23 months
Meal frequency (times/d)		6–23 months
Mean	2·0	
sd	0·8	
Met minimum meal frequency (%)	50	6–23 months
Met minimum acceptable diet (%)	7	6–23 months

#### Water, sanitation and hygiene practices

The main source of drinking-water for most (>90 %) of the households were boreholes. The majority (87 %) of the participants reported to have never treated water before drinking. One-quarter of the households did not have access to protected pit latrines (i.e. with a slab), which could be a medium for transmitting micro-organisms that cause pathogenic diarrhoea. Nearly 80 % of the children had experienced diarrhoea.

#### Aflatoxin contamination

Table 4 indicates that the children’s exposure to aflatoxin was largely varied. While half of the children had no or a non-detectable level of aflatoxin in urine (AFM1), the mean urine AFM1 level in 44 % of the samples was 45 pg/ml with the highest AFM1 level of 783 pg/ml. Aflatoxin contamination in the sampled crops was much less heterogeneous and contamination in many of the samples was below the critical level, although there were some high contamination cases. Against the odds, the *t* test did not show a significant relationship between aflatoxin contamination in food and urine (Table 5). Table 6 shows the difference in children’s undernutrition indicators by presence of AFM1 in urine. AFM1 in urine was significantly associated with wasting, but not with stunting and underweight. Albeit not presented, the same tendency was confirmed by least-squares regression analysis.

**Table 4:** Baseline aflatoxin concentration in maize and urine of the sampled children (*n* 241) from households in Mchiniji, Mzimba and Balaka districts, rural Malawi, 2014–2015

Variable	Mean	sd	Min.	Max.
Urine aflatoxin concentration (pg/ml)	45	89	0	783
Urine aflatoxin dummy (1 if above 1 pg/ml)	0·5	0·5	0	1
Maize aflatoxin concentration (ppb)	1·4	3·4	0	20·9
Maize aflatoxin dummy (1 if above 1 ppb)	0·25	0·4	0	1

**Table 5:** Mean difference in baseline aflatoxin concentration in urine (pg/ml) by maize contamination status in the sampled children (*n* 241) from households in Mchiniji, Mzimba and Balaka districts, rural Malawi, 2014–2015

Aflatoxin in food	*n*	Mean	sd	*t* statistic	*P* value
<1 ppb	180	44·8	75·8		
>1 ppb	61	46·7	120·6		
Difference		−2·0		−0·15	0·560

**Table 6:** Mean difference in baseline undernutrition indicators by urine aflatoxin status in the sampled children (*n* 241) from households in Mchiniji, Mzimba and Balaka districts, rural Malawi, 2014–2015

Malnutrition indicator	Aflatoxin in urine	*n*	Mean	sd	*t* statistic	*P* value
Weight-for-height	0 pg/ml	118	0·92	1·56		
*Z*-score (wasting)	>0 pg/ml	123	0·64	1·67		
	Difference		0·28		1·35	0·090
Height-for-age	0 pg/ml	118	−0·78	1·90		
*Z*-score (stunting)	>0 pg/ml	123	−0·60	1·80		
	Difference		−0·18		−0·77	0·779
Weight-for-age	0 pg/ml	118	0·16	1·38		
*Z*-score	>0 pg/ml	123	0·07	1·37		
(underweight)	Difference		0·09		0·50	0·308

#### Undernutrition status

Table 7 summarises the children’s anthropometric measurements and undernutrition indicators. The *Z*-scores of the sampled children were not notably inferior to the norm except for stunting. However, there was a non-negligible proportion of children who were severely undernourished. The MUAC was relatively homogeneous, averaged at 14 cm.

**Table 7:** Baseline undernutrition status of the children (*n* 241) from households in Mchiniji, Mzimba and Balaka districts, rural Malawi, 2014–2015

Anthropometric variable	Mean	sd	Min.	Max.
Weight (kg)	8·6	1·5	4·5	13·5
Height (cm)	69	5·4	56	84
Mid-upper arm circumference (cm)	14	1·2	10	17
Weight-for-height *Z*-score (wasting)	0·8	1·6	−4·6	6·2
Severe wasting dummy (1 if *Z*-score is below −2)	0·1	0·2	0	1
Height-for-age *Z*-score (stunting)	−0·7	1·8	−6·9	5·0
Severe stunting dummy (1 if *Z*-score is below −2)	0·2	0·4	0	1
Weight-for-age *Z*-score (underweight)	0·1	1·3	−5·0	3·3
Severe underweight dummy (1 if *Z*-score is below −2)	0·1	0·3	0	1

### Impacts of training on undernutrition status

Tables 8 shows the difference-in-difference estimation results of training impacts on wasting, stunting, underweight and MUAC. The models presented are fixed effect, random effect and ordinary least squares (OLS) with standard errors clustered on households.*

**Table 8:** Impacts of the training to mitigate child undernutrition on wasting, stunting, underweight and mid-upper arm circumference (MUAC) of children from households in Mzimba and Balaka districts, rural Malawi, 2014–2015: difference-in-difference estimations (DID; *n* 179)

	Weight-for-height *Z*-score (wasting)						Height-for-age *Z*-score (stunting)					
	Fixed effect		Random effect		OLS robust		Fixed effect		Random effect		OLS robust	
Dependent variable	Estimate	se	Estimate	se	Estimate	se	Estimate	se	Estimate	se	Estimate	se
Treatment dummy	NA		−0·70	0·085	−0·63	0·099	NA		0·59	0·093	0·67**	0·027
Day 7 dummy	0·01	0·952	0·01	0·952	0·19	0·411	−0·02	0·907	−0·02	0·907	0·14	0·487
Day 14 dummy	−0·16	0·445	−0·16	0·444	−0·13	0·610	0·05	0·714	0·05	0·714	0·18	0·368
Day 21 Dummy	−0·25	0·240	−0·25	0·238	−0·20	0·446	−0·04	0·763	−0·04	0·763	0·09	0·680
DID (day 7)	0·37	0·163	0·37	0·161	0·21	0·486	0·03	0·843	0·03	0·843	−0·14	0·521
DID (day 14)	0·71***	0·007	0·71***	0·006	0·72**	0·025	0·003	0·987	0·003	0·987	−0·14	0·561
DID (day 21)	0·85***	0·001	0·85***	0·001	0·84**	0·014	0·18	0·281	0·18	0·279	0·01	0·956
Constant	0·40***	0·000	0·87***	0·000	0·27	0·801	0·66***	0·000	0·26	0·363	−0·15	0·806
*F* statistic, *P* value	*F*_(6,153)_=3·71***, *P*=0·002				*F*_(14,52)_=1·91**, *P*=0·048		*F*_(6,153)_=0·51, *P*=0·801				*F*_(14,52)_=0·78, *P*=0·687	
Wald test *χ* ^2^, *P* value			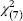 =22·6***, *P*=0·002						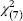 =6·72, *P*=0·458			
	Weight-for-age *Z*-score (underweight)						MUAC (cm)					
	Fixed effect		Random effect		OLS robust		Fixed effect		Random effect		OLS robust	
Dependent variable	Estimate	se	Estimate	se	Estimate	se	Estimate	se	Estimate	se	Estimate	se
Treatment dummy	NA		−0·25	0·365	−0·14	0·627	NA		0·33	0·609	0·36	0·189
Day 7 dummy	−0·03	0·853	−0·03	0·853	0·22	0·338	0·18	0·792	0·15	0·824	0·20	0·292
Day 14 dummy	−0·11	0·452	−0·11	0·451	0·01	0·971	0·12	0·860	0·12	0·859	0·25	0·343
Day 21 Dummy	−0·23	0·104	−0·23	0·102	−0·09	0·627	−1·62**	0·017	−1·62	0·016	−1·55	0·337
DID (day 7)	0·31*	0·065	0·31*	0·063	0·08	0·757	−0·17	0·833	−0·15	0·861	−0·21	0·413
DID (day 14)	0·53***	0·002	0·53***	0·002	0·44*	0·076	−0·02	0·979	−0·02	0·979	0·03	0·923
DID (day 21)	0·74***	0·000	0·73***	0·000	0·61**	0·013	1·92**	0·020	1·92**	0·019	1·94	0·253
Constant	0·62***	0·000	0·79***	0·001	0·31	0·638	0·12	0·655	−0·98	0·852	2·40	0·204
*F* statistic, *P* value	*F*_(6,153)_=5·91***, *P*=0·000				*F*_(14,52)_=2·66***, *P*=0·005		*F*_(6,152)_=1·72, *P*=0·120				*F*_(14,52)_=1·38, *P*=0·198	
Wald test *χ* ^2^, *P* value			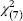 =35·78***, *P*=0·000						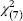 =14·03*, *P*=0·051			

NA, not applicable.

Control variables in the ordinary least squares (OLS) and random-effect regression models are not shown, which are mother’s age, household head’s sex, household head’s age, household head’s education, household size and asset holding. *P<0·10, **P<0·05, ***P<0·01.

For wasting, the impacts of the training were found to be insignificant on day 7 and significant on day 14 and day 21, with the largest magnitude observed on day 21. The result clearly indicates that the continued good practice enforced by the training led to alleviating the status of wasting among the children. The quantitative interpretation is that receiving the continued training raises the *Z*-score for wasting by 0·85 within 3 weeks, which appears to be a remarkable effect given the short period of time. Besides, all the children in the intervention group underwent a positive change in wasting during the 21d training.

By contrast, for stunting the impacts did not reach significance within the 21d time frame, which is comprehensible since height is much less variable than weight even for children in the growth phase. It should be noted, however, that the *P* value decreased largely from day 14 to day 21, suggesting that the effect would become significant over a slightly longer time horizon. In fact, a change was observed among moderately stunted children. Six out of the eleven children who had been moderately stunted at the beginning of the training registered improvement in their height during the 3-week training period.

For underweight, the impacts were similar to those in the case of wasting. The only difference is that the effect was already weakly significant on day 7, as indicated by the panel regression output. Quantitatively, receiving the continued training raised the *Z*-score for underweight by 0·73 on average, in a matter of 3weeks.

For MUAC, positive and significant impacts were found on day 21. Although there was no sign of improvement until day 14, the effect emanated in the third week of the intervention. Quantitatively, receiving the continued training raised the mean MUAC by 1·92 cm within 3weeks, although the growth was invisible until the second week of improved practice.

Apart from the impacts on the undernutrition indicators, the frequency of diarrhoea decreased significantly after the first week of the training only in the intervention group, indicating that the hygiene practices were effective as far as diarrhoea was concerned. Furthermore, the dietary diversity score increased from 2·0 to 4·0, suggesting the usefulness of the complementary food used in the intervention composed of diverse and nutritious ingredients such as legumes, coarse cereals, vitamin A-rich vegetables and green leafy vegetables.

Lastly and very importantly, the recommended recipes (Table 1) were met with the acceptability rate of 82 % in the intervention group. Children were particularly fond of Recipe 2 described in Table 1.

## Discussion

All recruited participants from both districts were engaged in maize production as maize is their staple food stored throughout the year. This explains why most of the infants and young children received whole-grain maize porridge as a complementary diet. The descriptive statistics also suggest that the sampled households were poor, and the dietary diversity was limited. The government and non-governmental organisations in Malawi have been promoting enriching maize flour porridge with other ingredients such as groundnut flour, soya flour, cooking oil and vegetables. However, child undernutrition rates remain unacceptably high, which implies that despite the promotion, adoption of diversified diets is slow, and that food safety and hygiene practices also need to be addressed, e.g. acute and chronic illnesses caused by poor hygiene, insufficient time taken by mothers on child care, and aflatoxin contamination.

Aflatoxin was detected in the urine samples of more than half of the children, while high contamination was found in maize samples from a quarter of the households. Given that there are two channels of aflatoxin absorption by children, i.e. complementary feeding and breast-feeding, our statistics suggest that there had been transmission of aflatoxin from mothers via breast-feeding as well. Moreover, consuming poor-quality food containing grade-outs as described by the study participants (Table 2) leads to increased aflatoxin exposure as likelihood of the presence of aflatoxin in grade-outs is high^(^ ^47^ ^)^. In the present study aflatoxin exposure among children was significantly associated with wasting status, which is consistent with a study conducted in West Africa^(^ ^37^ ^)^ that reported a strong association between aflatoxin exposure upon weaning and impaired growth of children.

At baseline, diarrhoea was rampant due to unclean water and other improper sanitation factors. Yet, the intervention contributed to quickly reducing the incidence of diarrhoea, which is regarded as an indirect cause for undernutrition. This indicates the effectiveness of the improved hygiene practices covered by the training.

The regression results proved the positive effects of the comprehensive training on the indicators of undernutrition among children, particularly wasting, underweight and MUAC. The 3-week training was conducive to increasing the *Z*-scores for wasting and underweight by 0·85 and 0·73, respectively, and MUAC by 1·92 cm on average. Albeit less significant, the result on stunting suggests a positive sign that behavioural changes arising from continuous feeding of a safe and balanced diet can also benefit height in a longer time frame. Although the health outcome of the training was positive, it is worth considering the acceptability of the recipe used. In our case, four out of five households in the intervention group accepted the recipe, indicating promising potential for upscaling similar capacity-building programmes. Nevertheless, there is still room for improvement in the recipe by incorporating the preferences of the remaining fifth, so that the knowledge dissemination would be sustainable.

There are three major limitations in the present study. First, since the study focused on the impacts of the comprehensive training, there was only one treatment group which received the entire package of intervention. In other words, the impact of a specific subject of training was not distinguished from that of another subject. Hence, there remains a question as to the extent to which each subject of training was effective and what synergy the combination of subjects brought forth. Second, since our focus was on the real-time impacts of the 3-week programme, longer-term impacts were beyond the scope of the study. The sustainability of the effects of the training would require further attention. These two remaining themes will be studied and documented in another article. Third, the blood aflatoxin–albumin biomarker is a more appropriate measure for chronic exposure status over the past few months^(^ ^48^ ^)^. In our case, however, the mothers were willing to provide urine samples only and hence the urine aflatoxin biomarker (AFM1) was employed, which is an indicator of 24 h exposure of aflatoxin contamination through food.

Another factor to note is that the present study employed common and conventional measures of undernutrition (wasting, stunting) and their composite (underweight). However, Nandy and Miranda^(^ ^7^ ^)^ argue that results on underweight can be contradictory to results on wasting and stunting, and suggest use of the Composite Index of Anthropometric Failure (CIAF) which is always consistent with the conventional indicators. Our result on underweight may alter to some extent with use of the CIAF. Nevertheless, in light of the consistency observed in our results between underweight and the two conventional indicators, the result and implication, arguably, would not change to a significant extent. Since the CIAF incorporates the failure in height-for-age which is not expected to change drastically within 3weeks, it may mask the short-term phenomena in our study.

Lastly, the children’s acceptability of Recipe 2 was high presumably due to the inclusion of pigeon pea and finger millet which were absent in Recipe 1. In particular, pigeon pea seemed to enhance the taste of the porridge in Recipe 2. The cereals and legumes chosen in Recipe 2 complement each other for protein and micronutrient requirements, which is absent in the usual maize porridge prepared by mothers. Details on the acceptance and nutrient contents of different recipes are beyond the scope of the present study, but will be illustrated in another ongoing study.

## Conclusion

Undernutrition in children has been detrimental to economic and social development in low-income countries, particularly sub-Saharan Africa. In an attempt to accumulate empirical evidence, the current paper offered the first quantitative estimation from Malawi of the impacts on undernutrition indicators of an intervention incorporating the PD/Hearth approach in which the mothers were educated on three subjects: diversified complementary diets, hygiene and food safety. A randomised controlled trial and difference-in-difference regression revealed significant improvement in health outcomes within 21d by virtue of the comprehensive training implemented. Moreover, the article presented the first results on aflatoxin assay of children’s urine in Malawi, showing that more than half of the sampled children were contaminated with aflatoxin.

The results unequivocally suggest that the comprehensive training immediately led to behavioural changes among mothers and that the improved practices took progressive effects in ameliorating children’s undernutrition. Direct observation of the immediate outcome will convince mothers to maintain the good practices that are affordable in the communities. Further elicitation and incorporation of local preferences and tastes would help guarantee sustainable adoption. In all likelihood, the result implies the need for policies and institutions to incentivise development agencies and governments to invest in upscaling intervention programmes of this sort targeted at relevant mothers in resource-poor rural communities.

## Footnotes

* The Hausman test pointed to random effects with all four dependent variables.

## References

[1] Lombard JM (2014) Mycotoxin exposure and infant and young child growth in Africa: what do we know? Ann Nutr Metab 64, 42–52. 25341872 10.1159/000365126

[2] National Statistical Office (2014) Malawi MDG (Millennium Development Goals) Endline Survey 2014, Key Findings. Zomba, Malawi: National Statistical Office.

[3] Mangili A, Murman DH, Zampini AM et al. (2006) Nutrition and HIV infection: review of weight loss and wasting in the era of highly active antiretroviral therapy from the nutrition for healthy living cohort. Clin Infect Dis 42, 836–842. 16477562 10.1086/500398

[4] Mitch WE & Goldberg AL (1996) Mechanisms of muscle wasting – the role of the ubiquitin–proteasome pathway. N Engl J Med 335, 1897–1905. 8948566 10.1056/NEJM199612193352507

[5] Smith LE, Stoltzfus RJ & Prendergast A (2012) Food chain mycotoxin exposure, gut health and impaired growth: a conceptual framework. Adv Nutr 3, 526–531. 22797988 10.3945/an.112.002188 PMC3649721

[6] Neumann CG & Harrison GG (1994) Onset and evolution of stunting in infants and children. Examples from the Human Nutrition Collaborative Research Support Program. Kenya and Egypt studies. Eur J Clin Nutr 48, 90–102. 8005095

[7] Nandy S & Miranda JJ (2008) Overlooking undernutrition? Using a composite index of anthropometric failure to assess how underweight misses and misleads the assessment of undernutrition in young children. Soc Sci Med 66, 1963–1966. 18299166 10.1016/j.socscimed.2008.01.021 PMC2685640

[8] Prasanna BM, Vasal SK, Kassahun B et al. (2001) Quality protein maize. Curr Sci 81, 1308–1319.

[9] Malawi Vulnerability Assessment Committee (2014) Market Assessment Report. Lilongwe: Government of Malawi.

[10] Messina AJ (1999) Legumes and soybeans: overview of their nutritional profiles and health effects. Am J Clin Nutr 70, Suppl. 3, 439S–450S. 10479216 10.1093/ajcn/70.3.439s

[11] Okpala LC & Okoli EC (2011) Nutritional evaluation of cookies produced from pigeon pea, cocoyam and sorghum flour blends. Afr J Biotechnol 10, 433–438.

[12] Kannan S (2010) Finger millet in nutrition transition: an infant weaning food ingredient with chronic disease preventive potential. Br J Nutr 104, 1733–1734. 20673383 10.1017/S0007114510002989

[13] Snapp SS & Fisher M (2015) ‘Filling the maize basket’ supports crop diversity and quality of household diet in Malawi. Food Sec 7, 83–96.

[14] Mlotha V, Mwangwela AM, Kasapila W et al. (2015) Glycemic responses to maize flour stiff porridges prepared using local recipes in Malawi. Food Sci Nutr 4, 322–328. 27004122 10.1002/fsn3.293 PMC4779488

[15] Wild CP & Gong YY (2010) Mycotoxins and human disease: a largely ignored global health issue. Carcinogenesis 31, 71–82. 19875698 10.1093/carcin/bgp264 PMC2802673

[16] Williams JH, Phillips TD, Jolly PE et al. (2004) Human aflatoxicosis in developing countries: a review of toxicology, exposure, potential health consequences, and interventions. Am J Clin Nutr 80, 1106–1122. 15531656 10.1093/ajcn/80.5.1106

[17] Turner PC (2013) The molecular epidemiology of chronic aflatoxin driven impaired child growth. Scientifica 2013, 152879. 24455429 10.1155/2013/152879 PMC3881689

[18] Williams JH, Grubb JA, Davis JW et al. (2010) HIV and hepatocellular and esophageal carcinomas related to consumption of mycotoxin-prone foods in sub-Saharan Africa. Am J Clin Nutr 92, 154–160. 20484447 10.3945/ajcn.2009.28761

[19] Keenan J, Jolly P, Preko P et al. (2011) Association between aflatoxin B1–albumin adduct levels and tuberculosis infection among HIV+ Ghanaians. Arch Clin Microbiol 2, issue 3, doi: 10:3823/230. PMC8486275 34603628

[20] Matumba L, Monjerezi M, Chirwa E et al. (2009) Natural occurrence of AFB1 in maize and effect of traditional maize flour production on AFB1 reduction in Malawi. Afr J Food Sci 3, 413–425.

[21] Monyo ES, Njoroge SMC, Coe R et al. (2012) Occurrence and distribution of aflatoxin contamination in groundnuts (*Arachis hypogaea* L) and population density of aflatoxigenic *Aspergilli* in Malawi. Crop Protect 42, 149–155.

[22] Atehnkeng J, Ojiambo PS, Cotty PJ et al. (2014) Field efficacy of a mixture of atoxigenic *Aspergillus flavus* Link:Fr vegetative compatibility groups in preventing aflatoxin contamination in maize (*Zea mays* L.). Biol Contr 72, 62–70.

[23] Cotty PJ, Bayman P, Egcl DS et al. (1994) Agriculture, aflatoxins, and *Aspergillus* . In The Genus Aspergillus: From Taxonomy and Genetics to Industrial Applications, FEMS Symposium No. 69, pp. 1–27 [KA Powell, A Renwick and JF Peberdy, editors]. New York: Plenum Press.

[24] Partnership for Aflatoxin Control in Africa (2013) PACA Strategy 2013–2022. Addis Ababa: PACA, African Union Commission.

[25] Velleman Y & Pugh I (2015) Under-Nutrition and Water, Sanitation and Hygiene. UK: WaterAid and SHARE (Sanitation and Applied Research for Equity consortium).

[26] Berggren G, Alvarez M & Genece E (1984) The Nutrition Demonstration Foyer: A Model for Combating Malnutrition in Haiti. Boston, MA: Massachusetts Institute of Technology.

[27] Zeitlin M, Ghassemi H & Mansour M (1990) Positive Deviance in Child Nutrition – With Emphasis on Psychosocial and Behavioural Aspects and Implications for Development. Tokyo: United Nations University.

[28] Bullen PB (2012) A multiple case study analysis of the positive deviance approach in community health. PhD Dissertation, Walden University.

[29] Bullen PAB (2011) The positive deviance/hearth approach to reducing child malnutrition: systematic review. Trop Med Int Health 16, 1354–1366. 21749582 10.1111/j.1365-3156.2011.02839.x

[30] Le Roux IM, le Roux K, Mbeutu K et al. (2011) A randomised controlled trial of home visits by neighborhood mentor mothers to improve children’s nutrition in South Africa. Vulnerable Child Youth Stud 6, 91–102. 22299019 10.1080/17450128.2011.564224 PMC3262232

[31] Ashworth A & Ferguson E (2008) Dietary counselling in the management of moderate malnourishment in children. Food Nutr Bull 30, 3 Suppl., S405–S433. 10.1177/15648265090303S304 19998865

[32] Nanchukwa KD (2007) Giving chance to indigenous knowledge in developing sustainable nutrition improvement interventions. In Proceedings of 2nd International AAAE Conference, Accra, Ghana, 18–22 August 2007, pp. 589–591. Nairobi: African Association of Agricultural Economists.

[33] Appoh LY & Krekling S (2005) Maternal nutritional knowledge and child nutritional status in the Volta region of Ghana. Matern Child Nutr 1, 100–110. 16881885 10.1111/j.1740-8709.2005.00016.x PMC6860941

[34] Marriott BP, White A, Hadden L et al. (2012) World Health Organization (WHO) infant and young child feeding indicators: associations with growth measures in 14 low-income countries. Matern Child Nutr 8, 354–370. 22171937 10.1111/j.1740-8709.2011.00380.x PMC6860880

[35] Brundtland GH (2000) Nutrition and infection: malnutrition and mortality in public health. Nutr Rev 58, 2 Pt 2, S1–S4. 10748611 10.1111/j.1753-4887.2000.tb07797.x

[36] Tchana AN, Moundipa PF & Tchouanguep FM (2010) Aflatoxin contamination in food and body fluids in relation to malnutrition and cancer status in Cameroon. Int J Environ Res Public Health 1, 178–188. 10.3390/ijerph7010178 PMC2819783 20195440

[37] Gong YY, Cardwell K, Hounsa A et al. (2002) Dietary aflatoxin exposure and impaired growth in young children from Benin and Togo: cross sectional study. BMJ 325, 20–21. 12098724 10.1136/bmj.325.7354.20 PMC116667

[38] Wright RC (1986) The seasonality of bacterial quality of water in a tropical developing country (Sierra Leone). J Hyg (Lond) 96, 75–82. 2869083 10.1017/s0022172400062550 PMC2129588

[39] Kennedy G, Ballard T & Dop M (2010) Guidelines for Measuring Household and Individual Dietary Diversity. Rome: FAO.

[40] Swindale A & Bilinsky P (2006) Household Dietary Diversity Score (HDDS) for Measurement of Household Food Access: Indicator Guide (v.2). Washington, DC: FHI 360/FANTA.

[41] Seetharaman N, Chacko TV, Shankar SLR et al. (2007) Measuring malnutrition – the role of Z scores and the composite index of anthropometric failure (CIAF). Indian J Community Med 32, 35–39.

[42] Blössner M & De Onis M (2005) *Malnutrition: Quantifying the Health Impact at National and Local Levels*. *WHO Environmental Burden of Disease Series* no. 12. Geneva: WHO.

[43] Nutrition Work Group, Child Survival Collaborations, and Resources Group (2002) Positive Deviance/Hearth: A Resource Guide for Sustainably Rehabilitating Malnourished Children. Washington, DC: CORE.

[44] Donald SG & Lang K (2007) Inference with difference-in-differences and other panel data. Rev Econom Stat 89, 221–233.

[45] Lechner M (2011) The estimation of causal effects by difference-in-difference methods. Found Trends Econom 4, 165–224.

[46] Tsusaka TW, Orr A, Msere HW et al. (2016) Do Commercialization and Mechanization of a ‘Women’s Crop’ Disempower Women Farmers? Evidence from Zambia and Malawi. Boston, MA: Agricultural & Applied Economics Association.

[47] Munthali WM, Charlie HJ, Kachulu L et al. (2016) How to Reduce Aflatoxin Contamination in Groundnuts and Maize: A Guide for Extension Workers. Patancheru, India: ICRISAT.

[48] Watson S, Gong YY & Routledge M (2015) Interventions targeting child undernutrition in developing countries may be undermined by dietary exposure to aflatoxin. Crit Rev Food Sci Nutr 57, 1963–1975. 10.1080/10408398.2015.1040869 26176888

